# Thymoquinone Enhances Doxorubicin Efficacy via RAS/RAF Pathway Modulation in Ovarian Adenocarcinoma

**DOI:** 10.3390/pharmaceutics17040536

**Published:** 2025-04-19

**Authors:** Veysel Toprak, İlhan Özdemir, Şamil Öztürk, Orhan Yanar, Yusuf Ziya Kizildemir, Mehmet Cudi Tuncer

**Affiliations:** 1 Department of Gynecology and Obstetrics, Faculty of Medicine, Private Metrolife Hospital, Şanlıurfa 63320, Turkey; drveysel21@outlook.com; 2 Department of Gynecology and Obstetrics, Faculty of Medicine, Atatürk University, Erzurum 25070, Turkey; ilhanozdemir25@yandex.com; 3 Vocational School of Health Services, Çanakkale Onsekiz Mart University, Çanakkale 17100, Turkey; ozturksamil@outlook.com; 4 Department of Gynecology and Obstetrics, Private Nev Hospital, Şanlıurfa 63300, Turkey; drorhan6532@outlook.com; 5 Department of Gynecology and Obstetrics, Şanlıurfa Training and Research Hospital, Şanlıurfa 63300, Turkey; dryusufziya34@outlook.com; 6 Department of Anatomy, Faculty of Medicine, Dicle University, Diyarbakir 21200, Turkey

**Keywords:** ovarian cancer, apoptosis, thymoquinone, immunofluorescent staining

## Abstract

**Background/Objectives:** Ovarian cancer remains one of the most commonly diagnosed malignancies among women worldwide. The heterogeneity among tumor subtypes and the emergence of treatment resistance have raised significant concerns regarding the long-term efficacy of chemotherapy, radiotherapy, and immunotherapy. In response to these challenges, drug repurposing strategies—utilizing existing drugs in novel therapeutic contexts—have gained increasing attention. This study aimed to investigate the cytotoxic and apoptotic effects of the combined application of doxorubicin (DX) and thymoquinone (TQ) on ovarian adenocarcinoma cells (OVCAR3). **Methods:** OVCAR3 cells were cultured in RPMI medium supplemented with 10% fetal bovine serum (FBS) and 1% penicillin/streptomycin. Cell viability and proliferation were assessed using the MTT assay following treatment with various concentrations of DX and TQ. NucBlue immunofluorescence staining was employed to examine nuclear morphology and to identify apoptosis-associated changes. Additionally, quantitative real-time polymerase chain reaction (qRT-PCR) was per-formed to evaluate the expression levels of apoptosis-related and oncogenic pathway genes, including RAF, RAS, Bcl-2, and Bax. **Results:** The results demonstrated that the combination of DX and TQ significantly reduced OVCAR3 cell viability and induced apoptosis in a dose-dependent manner. qRT-PCR analysis revealed a downregulation of RAS, RAF, and Bcl-2 expression, along with an upregulation of Bax, indicating activation of the intrinsic apoptotic pathway. These findings suggest that thymoquinone exerts an-ti-proliferative and pro-apoptotic effects by modulating the RAS/RAF signaling cascade. Furthermore, the co-administration of thymoquinone with doxorubicin potentiated these effects, suggesting a synergistic interaction between the two agents. **Conclusions:** Histopathological and molecular evaluations further confirmed the activation of apoptosis and the suppression of key oncogenic pathways. Collectively, these results underscore the therapeutic potential of thymoquinone as both a monotherapy and an adjuvant to conventional chemotherapy, warranting further validation in preclinical and clinical studies.

## 1. Introduction

Cancer is a multifactorial disease characterized by the uncontrolled and anarchic proliferation of certain normal cells in the body. These cells evade normal differentiation mechanisms, regulate their proliferation, and resist programmed cell death [1]. The activation of oncogenes and/or the inactivation of tumor suppressor genes leads to uncontrolled cell cycle progression and the disruption of apoptotic mechanisms [2]. For a normal cell to transform into a cancerous cell, it must accumulate specific physiological alterations, including sustained proliferative signaling, evasion of growth suppressors, resistance to cell death, limitless replicative potential, induction of angiogenesis, activation of invasion and metastasis, reprogramming of energy metabolism, evasion of immune system detection, tumor-promoting inflammation, genomic instability, and mutations [3].

Ovarian cancer is the fifth leading cause of cancer-related deaths in women [4]. It is classified based on the cell type from which the tumor originates and is divided into three main categories: epithelial, germ cell, and stromal tumors. Additionally, rare subtypes such as small cell carcinoma and sarcomas have been reported [5,6]. Epithelial ovarian cancer (EOC), which accounts for more than 85% of ovarian cancer cases, is responsible for the majority of ovarian cancer-related deaths. Most women with EOC are diagnosed at an advanced stage with metastatic disease, often characterized by extensive peritoneal carcinomatosis and abdominal ascites. Developing new methods for early diagnosis and treatment of this fatal disease could significantly reduce mortality rates [7].

The rat sarcoma virus (RAS) proteins are intracellular guanine nucleotide-binding proteins belonging to the family of small GTPases. These proteins play crucial roles in cell survival, cell cycle progression, cell polarity and movement, actin cytoskeleton organization, and vesicular and nuclear transport [8]. Among them, a proto-oncogene encoding a serine/threonine protein kinase (RAF) acts as an effector protein downstream of RAS. RAF promotes cell proliferation and survival by transmitting signals through the mitogen-activated protein kinase (MAPK) pathway. This signaling pathway functions downstream of various receptor tyrosine kinases, such as epithelial growth factor receptor (EGFR), and plays a key role in oncogenesis [9]. RAF mutations have been identified in 50% of malignant melanomas, 45% of papillary thyroid carcinomas, and 10% of colorectal cancers, as well as in ovarian, breast, and lung cancers [10].

Apoptosis, or programmed cell death, is a physiological process that involves cellular shrinkage, chromatin condensation, protein cleavage, DNA fragmentation, and phagocytosis, among other morphological and biochemical changes. It plays a crucial role in the morphological and functional development of multicellular organisms and is essential in the regulation of cancerous cells. The B-cell lymphoma 2 (Bcl-2) protein family plays a significant role in apoptosis, comprising both anti-apoptotic and pro-apoptotic molecules [11].

Currently, many plant-based treatments are being used as complementary therapies for cancer. Thymoquinone, a bioactive compound found in black cumin (*Nigella sativa*), is one such treatment [12]. Despite its reported antineoplastic, antibacterial, immunostimulatory, anti-inflammatory, and antioxidant properties, its exact mechanism of action remains unclear. However, studies have shown that thymoquinone induces apoptosis in cancer cells [13]. It has also been suggested that thymoquinone inhibits DNA synthesis in cancer cell lines and, due to its immunostimulatory and antioxidant properties, reduces oxidative stress and inflammation in tumor cells, thereby decreasing malignant transformation and enhancing natural killer cell activity [14,15].

Although both in vitro and in vivo experimental studies have demonstrated the inhibitory effects of thymoquinone and doxorubicin on cancer cell growth and progression, few studies have investigated whether these two agents promote cytotoxicity in ovarian adenocarcinoma cells. Therefore, the present study aims to investigate the effects of thymoquinone on the induction of apoptosis and the inhibition of proliferation in ovarian cancer cell lines under in vitro conditions.

## 2. Materials and Methods

### 2.1. Cell Culture

The OVCAR3 cell line (NIH: OVCAR-3, HTB-161™) was used in our laboratory. The cells were cultured in Roswell Park Memorial Institute (RPMI 1640) medium supplemented with 10% fetal bovine serum (Gibco, ThermoFisher, Waltham, MA, USA), 2 mM L-glutamine, and 1% penicillin/streptomycin. The cells were maintained as a monolayer culture in sterile culture dishes at 37 °C in a humidified environment containing 5% CO_2_ and 95% air. When the cells reached 80–90% confluence, they were passaged using ethylenediaminetetraacetic acid (EDTA) and trypsin. The detached cells were collected in a 15-mL centrifuge tube and centrifuged at 1000 rpm for 5 min. After the centrifugation process was completed, the supernatant was discarded, and the cell pellet was resuspended. A hemocytometer was used to determine the number of cells in the suspension obtained after centrifugation. A 10-μL aliquot of the suspended cells was removed and transferred to a 96-well plate. A 1:1 dilution was prepared by mixing 10 μL of the cell suspension with 10 μL of trypan blue. The number of viable cells in the hemocytometer was calculated using the following equation:

Number of cells per mL = Number of viable cells counted × Dilution factor × 10^4^ (1)

### 2.2. Determination of IC_50_

Stock solutions of doxorubicin and thymoquinone were prepared using pure ethanol. A 5 mM stock solution was prepared for doxorubicin, and a 50 mM stock solution was prepared for thymoquinone. During applications, the final concentration of the vehicle in the flasks or plate wells was reduced to 0.1%. To determine the inhibitory concentration (IC_50_) doses of doxorubicin and thymoquinone, the OVCAR-3 cell line was seeded into 96-well culture plates using an automatic multipipette at a density of 3 × 10^3^ cells per well. After overnight incubation (approximately 16 h), doxorubicin was applied at concentrations ranging from 0.5 to 50 µM, while thymoquinone was applied at concentrations ranging from 5 to 500 µM, using a total of nine different concentrations. The cells were then incubated for 24, 48, and 72 h.

### 2.3. MTT Assay

In the MTT analysis, the chemotherapy agent and control groups were designed to include six wells each. After incubation, cell viability analysis was performed. For this purpose, yellow tetrazolium MTT (3-(4,5-dimethylthiazolyl-2)-2,5-diphenyltetrazolium bromide) test solution was prepared at a concentration of 5 mg/mL and added to all wells at 20 µL per well. The plates were then incubated for 4 h. Following incubation, 200 µL of ultrapure dimethyl sulfoxide (DMSO) (Merck, Rahway, NJ, USA) was added to each well, and the plates were further incubated for 4 h in the dark. At the end of this period, absorbance readings were taken spectrophotometrically at wavelengths of 492, 570, and 650 nm. The viability of the control group was set as 100%, and the viability rates of the experimental groups were determined in comparison to the control. IC_50_ values for each cell line and chemotherapy agent in both the control and experimental groups were calculated using probit analysis with the SPSS 20 statistical package program.

### 2.4. Metastasis Analysis

The wound healing assay, used to study cell migration and cell–cell interactions, was performed to evaluate the effects of single and combined doses of TQ and DX on OVCAR-3 cells. For the assay, cells were seeded in a 6-well plate and cultured until they reached 100% confluence. Once the cells adhered to the plate surface, the medium was removed. A scratch was then created using a 200-µL pipette tip to generate a cell-free area, allowing for the observation of cell migration and gap closure. Following the scratch, the wells were washed with PBS, and the designated dose groups were applied in fresh medium. The study was terminated at the 36th hour, when the scratch in the control group was completely closed. At this point, the cells were photographed, and the images were analyzed by comparing them with the control group.

### 2.5. Total RNA Isolation

OVCAR-3 cells were incubated until they reached the logarithmic phase. Once the cells reached this phase, control, doxorubicin IC_50_: 2.12 μM, and thymoquinone IC_50_: 62.9 μM doses were applied individually. RNA was isolated from the samples 48 h after agent treatment. During the isolation phase, the PureLink RNA Mini Kit (Thermo, ThermoFisher, Waltham, MA, USA) was used, following the kit protocol. According to the protocol, 1% mercaptoethanol was added to the lysis solution provided in the kit. The culture medium was removed, and 1 mL of this lysis solution was added to each 25-mL flask that had been previously washed with D-PBS. These flasks were then incubated at 37 °C for 20 min, with gentle manual shaking every 5 min. After incubation, the lysed cells were collected in 2-mL Eppendorf tubes, and an equal volume of 1 mL of 70% ultrapure ethanol (Merck, USA) was added. The mixture was vortexed and then loaded onto the columns provided in the kit in 700-µL aliquots. The samples were sequentially centrifuged at 12,000× *g* for 30 s, allowing the RNA to bind to the column. The columns were then washed, first with washing solution 1, followed by two washes with washing solution 2, each time centrifuging at 12,000× *g* for 30 s. After the washing steps, the columns were centrifuged again at 12,000× *g* for 3 min to ensure they were completely dry. The columns were then transferred to new sterile 1.5-mL Eppendorf tubes, and 60 µL of the elution solution provided in the kit was pipetted directly onto the center of the membrane. The RNA was eluted by centrifugation at 12,000× *g* for 1 min, and the purified RNA was collected in Eppendorf tubes. The purity of the collected RNA samples was determined using an Optizen NanoQ microvolume spectrophotometer (Mecasys, Daejeon, Republic of Korea), and all samples were normalized to a concentration of 750 ng/10 µL with ultrapure water.

### 2.6. cDNA Synthesis

cDNA synthesis was performed to enable the amplification of the RNA obtained after the synchronization process by PCR. At this stage, the High-Capacity cDNA Reverse Transcription Kit (Life Technologies, Carlsbad, CA, USA) was used, following the kit protocol. The enzyme, dNTP mix, and random primers provided in the kit were mixed and pipetted into PCR tubes at a volume of 10 µL per tube. Subsequently, the total RNA, standardized to 750 ng/10 µL as described in the previous section, was added to the same tubes. The tubes were then incubated in the Applied Biosystems ProFlex PCR System thermal cycler using the following program:

- Step 1: 25 °C for 10 min
- Step 2: 37 °C for 120 min
- Step 3: 85 °C for 5 min

Following cDNA synthesis, the obtained cDNA samples were stored at −20 °C for further analysis.

### 2.7. Quantitative Real-Time PCR

The expression levels of the RAS, RAF, Bcl2 and Bax genes treatment groups of OVCAR-3 cells were analyzed by the qRT-PCR method. The primers of these genes are given below in the order 5′-3′.

- RAS: F: ACAGAGAGTGGAGGATGCTTT, R: TTTCACACAGCCAGGAGTCTT
- RAF: F: GGGAGCTTGGAAGACGATCAG, R: ACACGGATAGTGTTGCTTGTC
- BCL-2: F: ATGTGTGTGGAGAGCGTCAA, R: ACAGTTCCACAAAGGCATCC
- BAX: F: TTCATCCAGGATCGAGCAGA, R: GCAAAGTAGAAGGCAACG
- β-Actin: F: CCTCTGAACCCTAAGGCCAAC, R: TGCCACAGGATTCCATACCC
- GAPDH: F:CGGAGTCAACGGATTTGGTCGTAT, R: GCCTTCTCCATGGTGGTGAAGAC

The cDNAs obtained from RNA isolation were used for gene expression analysis. These cDNAs were analyzed by qRT-PCR following the Power SYBR Green qPCR Master Mix (Thermo, USA) protocol. The amplification process was performed using the Applied Biosystems QuantStudio 5 Real-Time PCR system.

The qRT-PCR reaction was carried out under the following conditions:

- Step 1: Enzyme activation at 95 °C for 10 min
- Step 2: Denaturation at 95 °C for 15 s, followed by primer binding and chain extension at 60 °C for 1 min
- Step 3: Melting curve analysis at 95 °C for 15 s, 60 °C for 1 min, and 95 °C for 15 s

The Ct values obtained from the amplification process were used to determine gene expression levels, which were calculated using the 2^−∆∆Ct^ method. Endogenous controls GAPDH (glyceraldehyde-3-phosphate dehydrogenase) and β-actin mRNA expressions were used for calibration and normalization, following the multiple control method.

### 2.8. Protein–Protein Interaction (PPI) Analysis

PPI data were retrieved from the STRING database. The STRING database provides information on protein–protein interactions (PPIs), along with confidence scores for data reliability. A confidence score of 0.4 or higher was selected to construct the interaction network of proteins associated with the target genes.

### 2.9. Enrichment Analysis

Data on the functional annotation of genes and the canonical pathways associated with the strong connections established with these proteins were obtained using the ShinyGO 0.80 program.

### 2.10. GO Functional Enrichment Analysis

Three types of gene ontology (GO) analyses were performed on potential target genes: cellular component (CC), biological process (BP), and molecular function (MF). The SRplot bioinformatics program was used to evaluate these data.

### 2.11. Statistical Analysis

The differences between the mean cell viabilities determined by the MTT assay and the expression values obtained from qRT-PCR studies were analyzed using one-way ANOVA. The Tukey HSD test was used to determine which groups differed significantly. Comparisons between two groups were performed using either the independent sample *t*-test or the Mann-Whitney U test, depending on the homogeneity of the data. Statistical analyses were conducted using the SPSS 20 (IBM, Armonk, NY, USA) program, with a significance level of *p* ≤ 0.05.

## 3. Results

### 3.1. Thymoquinone Modulates the RAS/RAF/MEK/ERK Pathway to Inhibit Proliferation and Induce Apoptosis in Cancer Cells

**Figure 1:**
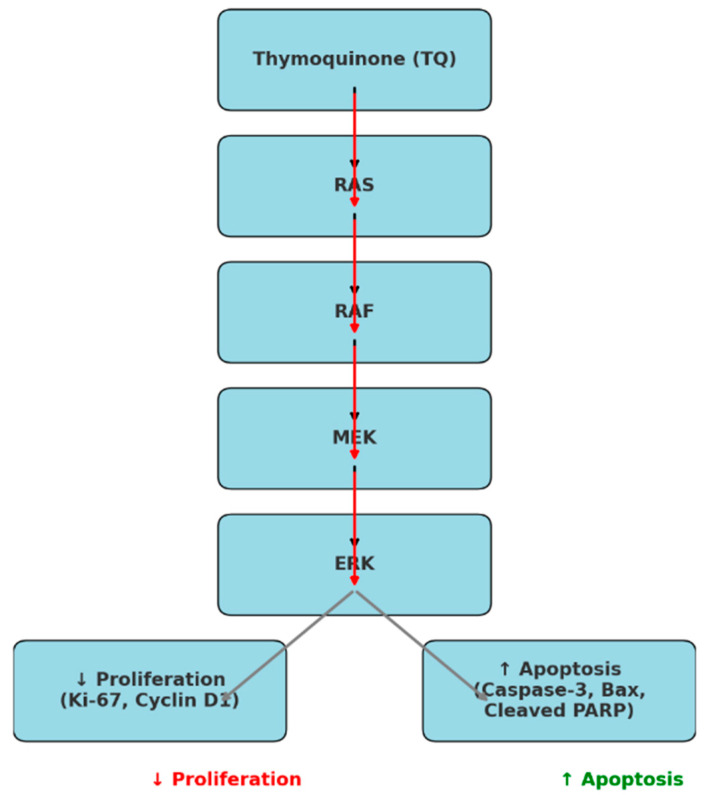
The inhibitory effect of thymoquinone on the RAS/RAF/MEK/ERK signaling pathway. TQ suppresses ERK activation, leading to decreased proliferation (represented in red) and increased apoptosis (represented in green). The downregulation of proliferation markers Ki-67 and Cyclin D1 (red) indicates reduced tumor cell growth, while the upregulation of apoptotic markers Caspase-3, Bax, and Cleaved Poly(ADP-ribose) polymerase (PARP) (green) promotes programmed cell death. This suggests that TQ exerts its anticancer effects by disrupting the MAPK signaling cascade, ultimately shifting the balance from cancer cell survival to apoptosis.

Our study demonstrates that TQ modulates the RAS/RAF/MEK/ERK signaling pathway, a key regulator of cancer cell proliferation and survival, in OVCAR-3 cells. The results indicate that TQ treatment leads to a reduction in proliferation markers (Ki-67, Cyclin D1) and an increase in apoptotic markers (Caspase-3, Bax, Cleaved PARP), confirming its role in apoptosis induction. This effect is further enhanced when combined with doxorubicin. By disrupting ERK activation, TQ shifts the balance toward apoptosis, reducing cell viability and migration, as evidenced by MTT and wound healing assays. These findings highlight TQ’s potential as an adjuvant therapeutic agent in ovarian cancer treatment (Figure 1).

### 3.2. Caspase-3 Mediated PARP Cleavage: A Key Mechanism in DNA Damage-Induced Apoptosis

**Figure 2:**
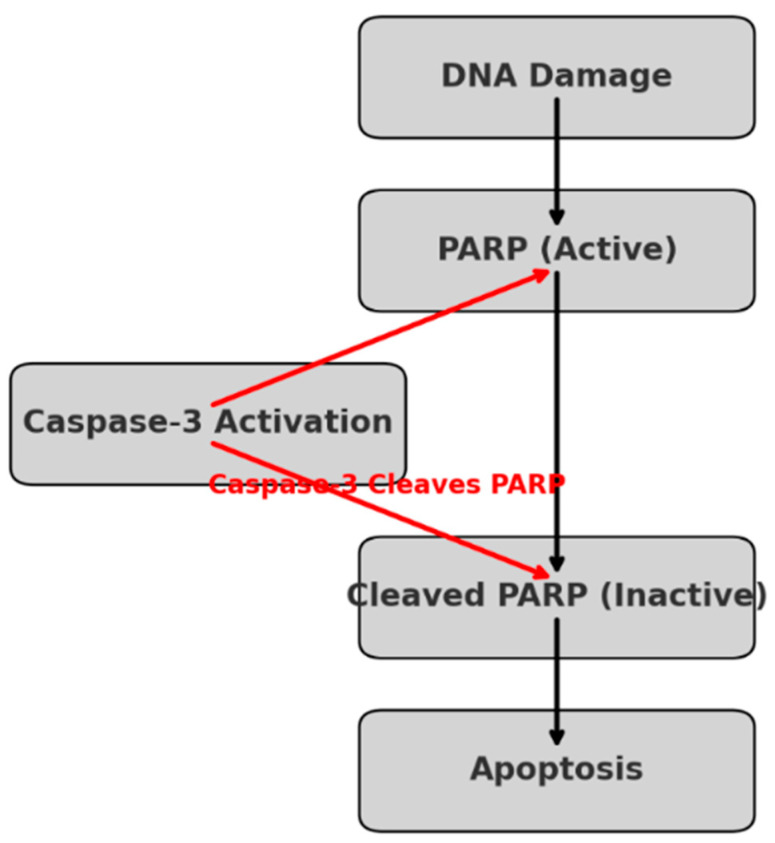
The role of PARP (Poly(ADP-ribose) polymerase) in apoptosis following DNA damage. When DNA damage occurs, active PARP attempts to repair the damage. However, if apoptosis is initiated, Caspase-3 activation leads to PARP cleavage, converting it into its inactive (cleaved) form. This prevents DNA repair, committing the cell to apoptosis. The red arrows indicate the role of Caspase-3 in cleaving PARP, a key step in apoptotic progression.

Our study confirms that PARP cleavage plays a significant role in TQ-induced apoptosis in OVCAR-3 cells. The results show that TQ treatment leads to increased Caspase-3 activation, which in turn cleaves PARP, rendering it inactive and preventing DNA repair. This process commits the cancer cells to apoptosis, as evidenced by increased levels of Cleaved PARP detected in our molecular analysis. The observed reduction in cell viability and enhanced apoptosis, particularly in the TQ + DX group, further supports this mechanism. These findings highlight the potential of TQ in sensitizing ovarian cancer cells to apoptosis through PARP cleavage, providing a mechanistic basis for its therapeutic potential (Figure 2).

### 3.3. OVCAR-3 Findings

**Figure 3:**
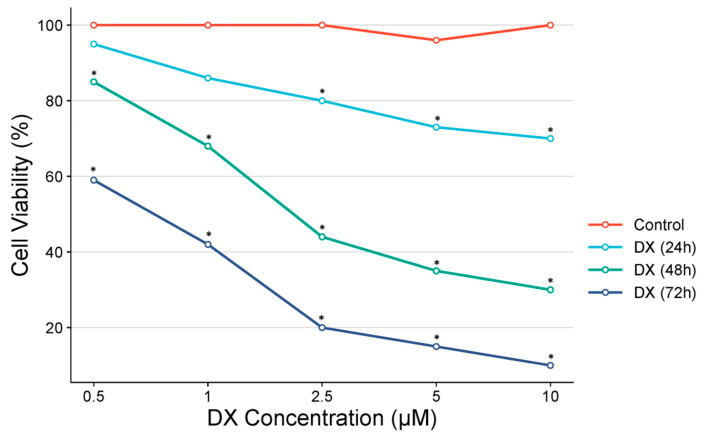
Percentage of cell viability in the OVCAR-3 cell line following doxorubicin treatment, along with IC_50_ values calculated using probit analysis, based on MTT assay results (n = 6; data are mean ± standard deviation values, IC_50_ values were calculated by probit analysis).* Data are statistically significant compared to control, one-way ANOVA, Tukey HSD test, *p* < 0.05.

In the MTT test performed on OVCAR-3 cells by administering different doses of DX, the IC_50_ value could not be found because cell proliferation did not fall below 50% in 24-h cell viability. However, after 2.5 µM, a statistically significant difference was detected compared to the control group. IC_50_ at the 48th hour was determined as 2.12 and IC_50_ at the 72nd hour was determined as 0.08 µM. A statistically significant difference was detected compared to the control group after 0.5 µM in 48 and 72 h of DX treatment (Figure 3).

**Figure 4:**
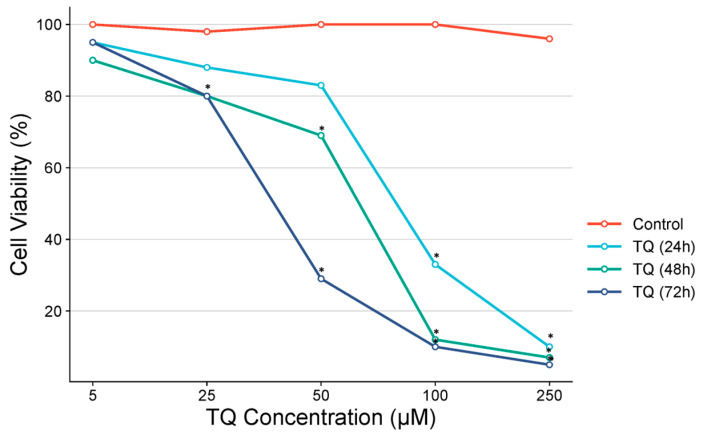
Cell viability percentages in the OVCAR-3 cell line were assessed after thymoquinone treatment using the MTT assay, and IC_50_ values were determined through probit analysis. (n = 6; data are mean ± standard deviation values, IC_50_ values were calculated by probit analysis).* Data are statistically significant compared to control, one-way ANOVA, Tukey HSD test, *p* < 0.05.

In the TQ-treated group, the IC_50_ value at 24 h was determined as 97.9 µM, at 48 h as 62.9 µM, and at 72 h as 37.5 µM. A statistically significant difference compared to the control group was observed at concentrations above 75 µM for 24-h TQ treatment, above 50 µM for 48-h TQ treatment, and above 10 µM for 72-h TQ treatment. The effects of TQ and DX at varying dose ranges and time points are shown in Figure 4.

### 3.4. Cell Migration Findings

**Figure 5:**
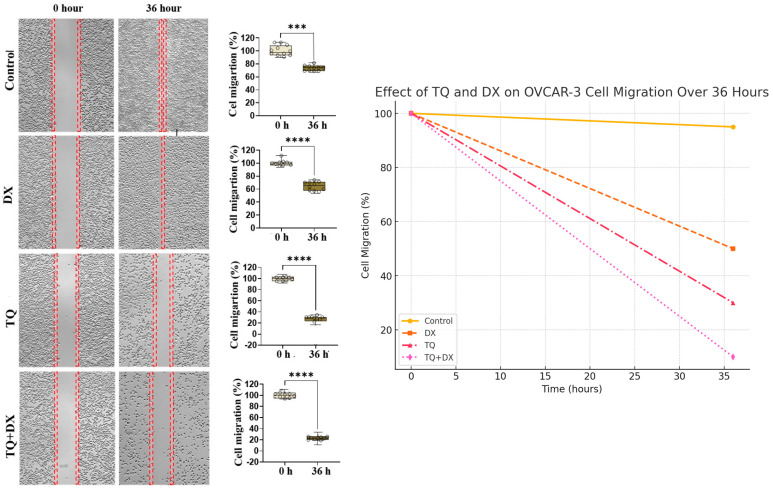
Cell migration rate and percentage of wound closure determined by the wound healing assay over 36 h in OVCAR-3 cell populations. n = 12; bars represent the mean ± standard error. Data are statistically significant, calculated using the independent samples *t*-test, *** *p* ≤ 0.001, **** *p* ≤ 0.0001. The curve graph further illustrates the progressive decrease in cell migration across different treatment groups over 36 h. The control group exhibited minimal reduction in migration, whereas DX treatment moderately inhibited migration. TQ treatment significantly reduced migration, and the TQ + DX combination resulted in the most substantial inhibition, blocking cell migration almost entirely. The red dashed lines in the wound healing images indicate the initial wound area at 0 h and serve as a reference to assess the extent of wound closure over 36 h. In the control group, the gap is nearly closed, demonstrating high cell migration.

In the cell migration assay, the wound area in the control group was completely closed by 36 h (100%), whereas the healing rate in the group treated with doxorubicin (DX) alone was approximately 50%. In the group treated with thymoquinone (TQ) alone, about 20% of the wound area was closed, while the TQ + DX combination treatment group exhibited a wound closure rate of only around 10%. Statistical analysis of cell migration rates revealed a highly significant difference between the treatment groups (TQ, DX, TQ + DX) and the control group (*p* ≤ 0.001). Furthermore, when the treatment groups were compared with each other, a significant difference was observed between the DX and TQ groups (*p* < 0.01), between DX and TQ + DX (*p* ≤ 0.001), and between TQ and TQ + DX (*p* < 0.05). These results indicate that the combination treatment most effectively inhibited cell migration (Figure 5).

However, in the treatment groups, especially TQ + DX, the persistence of the gap signifies a strong inhibition of cell motility, further confirming the anti-migratory effects of the treatments. These findings highlight the synergistic effects of TQ and DX in suppressing ovarian cancer cell motility.

### 3.5. Immunofluorescent Nucblue Staining Findings

**Figure 6:**
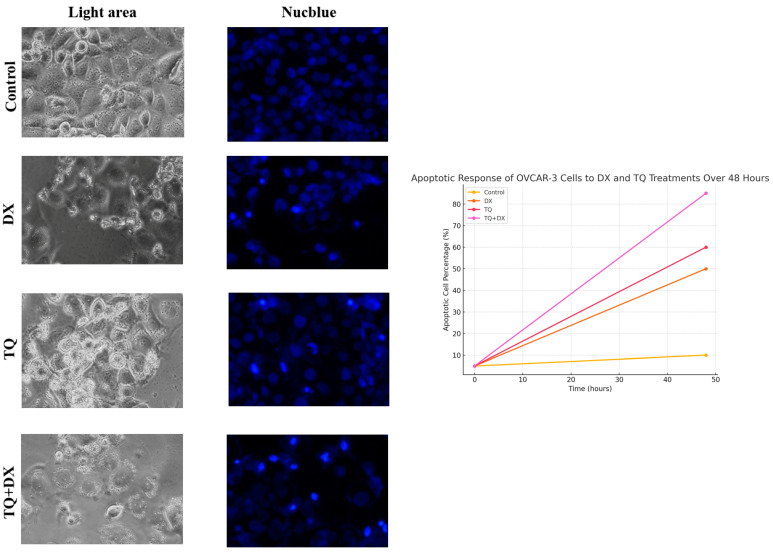
Phase-contrast (light area) and fluorescence (NucBlue staining) microscopy images of OVCAR-3 ovarian adenocarcinoma cells under different treatment conditions: control (untreated), DX, TQ, and the combination of TQ and DX. In the light area images, the control group shows a normal adherent morphology with intact cell membranes, while the DX and TQ groups exhibit signs of apoptosis such as cellular shrinkage, rounding, and detachment. The TQ + DX group demonstrates the most pronounced apoptotic features, including membrane blebbing and increased cell detachment. In the NucBlue-stained images, the control cells display uniformly stained nuclei, whereas the DX and TQ groups show nuclear fragmentation and chromatin condensation, indicative of apoptosis. The TQ + DX group exhibits the highest level of nuclear condensation and apoptotic body formation, suggesting an enhanced apoptotic effect. These findings confirm that both DX and TQ induce apoptosis in OVCAR-3 cells, with the combination treatment exerting the strongest effect, indicating a potential synergistic mechanism in promoting cell death.

Immunofluorescent NucBlue staining was performed to monitor the apoptotic process and assess the apoptotic effects of the treatment agents. While the cell morphology and nuclear structure appeared normal in the control group, a bright fluorescence signal was observed in the TQ- and DX-treated cells, indicating nuclear degradation. The highest level of apoptosis was detected in the TQ + DX combination treatment group (Figure 6).

### 3.6. Effect of Thymoquinone (TQ) and Doxorubicin (DX) on the mRNA Expression Levels of RAS, RAF, BCL-2, and BAX in OVCAR-3 Cells

**Figure 7:**
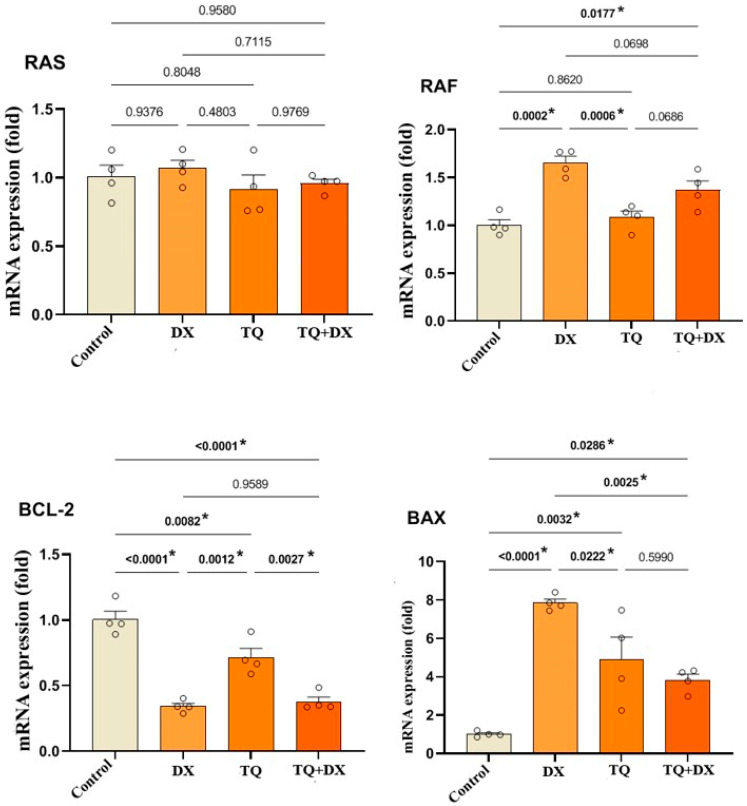
In the OVCAR-3 cell line, DX IC_50_: 2.12 μM, TQ IC_50_: 62.9 μM, relative fold increase values of RAS, RAF, Bcl2 and Bax gene expressions 48 h after single and combined drug application (n = 4 data mean ± SH), * means are statistically different, one-way ANOVA, Tukey HSD test.

When the expression results of the RAS gene, which plays a crucial role in proliferation, were evaluated, no significant difference was observed between the control and treatment groups (Figure 7). In contrast, a significant change in RAF gene expression was observed between the control and the DX and TQ + DX treatment groups, while no significant difference was found between the control and TQ treatment groups (Figure 7). Similarly, when the expression levels of apoptosis-regulating genes were analyzed, Bcl2 expression showed a significant decrease in all treatment groups compared to the control, with the most pronounced reduction observed in the DX and TQ + DX treatment groups. A statistically significant difference was also detected in Bax gene expression among all treatment groups compared to the control. The highest increase in Bax expression was observed in the DX group, followed by the TQ and TQ + DX treatment groups, respectively (Figure 7).

### 3.7. PPI Analysis

**Figure 8:**
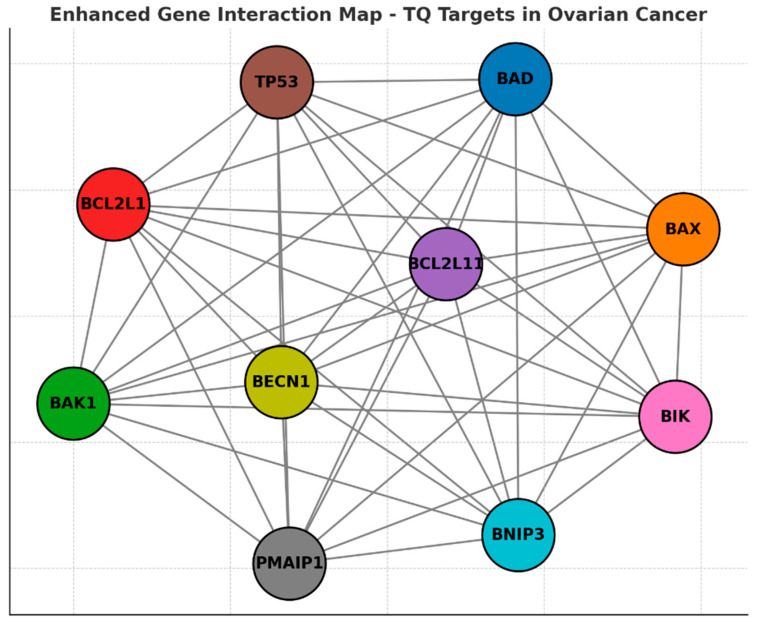
Protein–protein interactions (PPI) and interaction between various genes of ovarian cancer. This network visualization represents the gene interaction map of thymoquinone targets in ovarian cancer. The nodes correspond to key genes involved in apoptosis and cell survival, while the edges indicate PPI predicted through STRING analysis. The top 10 central genes, including TP53, BAD, BAX, BCL2L1, BCL2L11, BAK1, BIK, PMAIP1, BECN1, and BNIP3, are highlighted with distinct colors. These genes play critical roles in apoptotic regulation, mitochondrial integrity, and tumor progression. The highly interconnected structure suggests that TQ modulates multiple signaling pathways, reinforcing its potential as an anticancer agent targeting ovarian cancer cells.

Predictions from the STRING analysis were used to illustrate protein interactions. The visualization identified 11 nodes and 46 edges (Figure 8). Based on nodal degree, the following genes were identified as the top 10 central genes: BAD, BAX, BAK1, BCL2L1, BCL2L11, TP53, BIK, PMAP1, BECN1, and BNIP3. These genes are hypothesized to be the primary targets of TQ in ovarian cancer.

### 3.8. KEGG Pathway Enrichment Analysis

**Figure 9:**
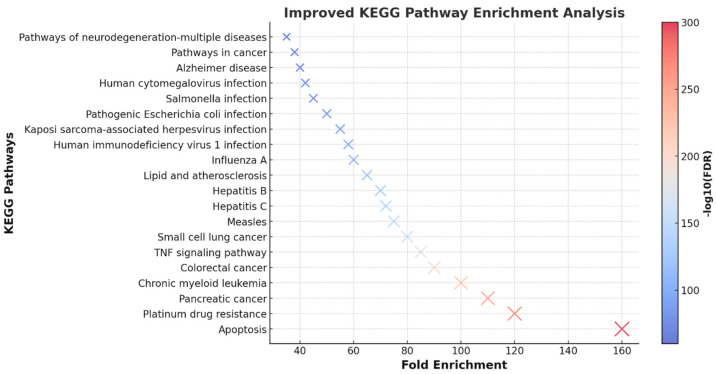
Enrichment analysis for the 123 common compound targets. This scatter plot represents the KEGG pathway enrichment analysis based on fold enrichment, number of genes, and −log10(FDR) values. Each dot corresponds to a significantly enriched pathway, with dot size representing the number of genes involved and color intensity indicating statistical significance (−log10(FDR)). The most enriched pathways include apoptosis, platinum drug resistance, and pancreatic cancer, suggesting key mechanisms influenced by thymoquinone in ovarian cancer. This analysis highlights crucial molecular pathways that may play a role in disease progression and therapeutic response.

KEGG pathway enrichment analysis of target genes was performed using the Shiny 0.80 program. The findings showed that 123 genes were involved in the enrichment process and 75 pathways were cancer-related, exhibiting a significant correlation with target genes (*p* < 0.05). Apoptosis, platinum drug resistance, pancreatic cancer, chronic myeloid leukemia, colorectal cancer, the tnf signaling pathway, small cell lung cancer, measles, hepatitis C, hepatitis B, the top 10 pathways that occur in diabetic complications, are shown (Figure 9).

### 3.9. GO Functional Enrichment Analysis Findings

**Figure 10:**
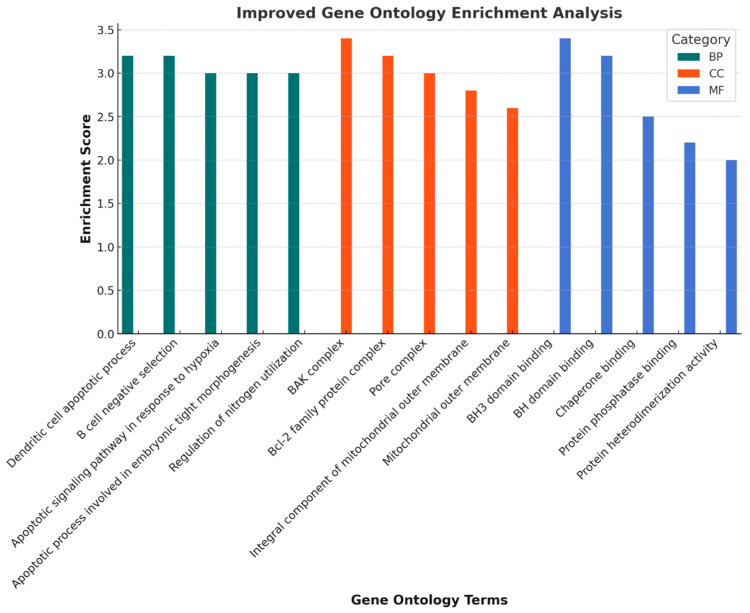
Gene ontology (GO) enrichment analysis of TQ targets in ovarian cancer. This bar chart illustrates the enrichment scores of biological processes (BP), cellular components (CC), and molecular functions (MF) associated with TQ target genes in ovarian cancer. The highest enriched terms include “BAK complex” and “BH3 domain binding”, highlighting their potential involvement in apoptotic regulation. BP terms (teal) indicate processes such as apoptotic signaling and hypoxia response, while CC terms (orange) focus on mitochondrial and protein complexes. MF terms (blue) reveal key molecular interactions related to protein binding. This analysis provides insights into the functional roles of TQ in ovarian cancer progression and treatment strategies.

Analysis findings show only important functions. Target genes were found to be involved in various cellular components in the BP category, such as the apoptotic process. In terms of cellular components, the BAK complex, and the Bcl2-family complex, it was found that the MF category exhibited roles such as BH3 domain binding, chaperone binding, and protein phosphatase binding (Figure 10).

## 4. Discussion

Ovarian cancer remains a significant global health concern, with high mortality rates primarily due to late-stage diagnosis, metastasis, and the emergence of chemoresistance. Conventional treatment strategies, including chemotherapy, radiotherapy, and immunotherapy, have shown limited success in overcoming drug resistance, necessitating the exploration of alternative therapeutic agents. In this context, thymoquinone, a bioactive compound derived from *Nigella sativa*, has gained attention for its anticancer properties, particularly its ability to modulate key oncogenic pathways, induce apoptosis, and inhibit proliferation.

Our study provides novel insights into the molecular mechanisms underlying TQ’s anticancer effects in OVCAR-3, specifically through its modulation of the RAS/RAF/MEK/ERK signaling pathway. The combination of TQ with doxorubicin significantly enhanced cytotoxicity and apoptosis, suggesting a potential synergistic effect. Importantly, our findings reveal that TQ not only promotes apoptosis by increasing pro-apoptotic markers (Bax, Caspase-3, Cleaved PARP) and reducing anti-apoptotic markers (Bcl-2) but also demonstrates anti-migratory effects, as evidenced by wound healing assays. The observed suppression of metastatic potential further highlights TQ’s therapeutic promise as an adjuvant agent in ovarian cancer treatment.

These results align with previous reports suggesting that TQ exerts anticancer activity by targeting multiple cellular processes, including oxidative stress reduction, mitochondrial dysfunction, and immune modulation. The ability of TQ to disrupt cancer cell survival mechanisms, either alone or in combination with standard chemotherapeutic agents, positions it as a potential candidate for overcoming chemoresistance and enhancing therapeutic efficacy in ovarian cancer. However, further in vivo studies and clinical trials are necessary to validate its clinical applicability and establish optimal treatment protocols for its use in combination therapies.

In vitro studies have shown that the essential oils found in black cumin seeds have cytotoxic effects against different human cancer cell lines. Thymoquinone is also cytotoxic for human cancer cell lines such as colorectal, pancreatic adenocarcinoma, uterine sarcoma, and leukemia [16,17]. In our study, in cell culture experiments, it was determined that all dilutions of thymoquinone up to 75 µM dilution were not cytotoxic on OVCAR3 cells when applied for 24 h. However, it was reported that it inhibited the proliferation of OVCAR3 cells above this dose. Cytotoxicity also increased, especially with increasing dose-related time. The antitumor mechanisms of *Nigella sativa* have been shown in various studies [17]. Depending on its concentration, *Nigella sativa* extracts have been reported to exhibit antitumor activity by inhibiting metastasis-stimulating factors, including type IV collagenase, matrix metalloproteinases, angiogenic proteins such as fibroblast growth factor, tissue-type plasminogen activator, urokinase-type plasminogen activator, plasminogen activator inhibitor type 1, and serine protease inhibitors [18,19,20]. To examine the effects of thymoquinone and doxorubicin, alone or in combination, on the migration of OVCAR3 cells, a wound healing assay was performed, and the results are described above. IC_50_ values obtained by the MTT test were used to observe the effect of drugs on cell migration. According to the results of the cell migration assay, doxorubicin and thymoquinone, alone and in combination, inhibited the migration of OVCAR3 cells in a time- and dose-dependent manner after 36 h. Finally, the greatest inhibition of OVCAR3 cell migration was determined in the combination treatment. Similar studies for thymoquinone have shown that it inhibits the migration of different cancer cells, but we did not find any studies of the effect of its combination with doxorubicin on ovarian cancer cells [21]. In this study, its reaction upon wound healing after 36 h also revealed its metastatic role. It is also suggested that thymoquinone may have an antineoplastic effect, exerted by regulating antitumor immune responses [22].

TQ belongs to a family of quinones that can undergo enzymatic or non-enzymatic redox cycling with semiquinone radicals to form superoxide anion radicals. It has proven its effectiveness against various diseases, thanks to its many medical and pharmacological activities, such as anti-inflammatory, antioxidant, hepatoprotection, neuroprotector, histone protein modulator, insecticidal, anti-ischemic, and radioprotector effects. TQ differentially activates a variety of molecular targets, and its effects are mediated by a variety of cellular mechanisms, including proliferation inhibition, induction of apoptosis, cell cycle disruption, production of reactive oxygen species (ROS), and inhibition of angiogenesis and cellular metastasis [23,24]. Thymoquinone also interferes with the structure of DNA. It targets cellular copper, which is found in chromatin and is strongly associated with DNA-based guanine, resulting in DNA oxidation and cancer cell death. It may also have an effect on DNA synthesis in cancer cells. It also inhibits the proliferation and migration of human non-small cell lung cancer by reducing ERK1/2 phosphorylation [17,25]. In addition to its inhibitory effect on cell proliferation and survival, TQ also promotes the apoptosis of cancer cells. According to numerous studies, TQ is thought to cause intrinsic apoptotic cell death by reducing the expression of the anti-apoptotic protein family BCL2 and increasing mitochondrial-dependent caspase activation [26]. Thymoquinone has demonstrated anticancer activity in various in vitro and in vivo studies, as well as in adjuvant settings to prevent carcinogenesis or enhance the efficacy of conventional therapeutic approaches. Doxorubicin, for instance, is associated with several adverse effects, occurring in more than 10% of patients. In some cases, thymoquinone has been shown to mitigate the harmful effects of doxorubicin, while exhibiting a broad spectrum of bioactivity [27].

As a result, thymoquinone slows down reproduction in the ovarian cancer cell line, in many different types of cancer, indicating that it is a strong chemical protector that protects DNA [28,29]. A similar situation has been observed for forestomach fibrosarcoma, colon, skin and liver tumors and it has been suggested to be a potent chemo protectant [19,30]. Ovarian cancer remains a significant public health issue affecting women across all populations. The findings of this study suggest that the combination of TQ and DX may play a pivotal role in the treatment of ovarian cancer by inducing apoptosis. The cytotoxic effects of TQ and DX were evaluated using both the OVCAR-3 ovarian cancer cell line and the HaCaT healthy keratinocyte cell line, representing a novel aspect of this research [31]. Based on the data obtained, study parameters were expanded, and detailed insights into the apoptotic pathway were achieved. In addition to assessing cell viability and proliferation using the MTT assay, cell migration was evaluated, apoptotic cell death was detected via immunofluorescence staining, and the expression levels of RAS/RAF and Bcl-2 genes were analyzed using qRT-PCR. These findings underscore the importance of future studies. We propose that thymoquinone is a promising agent for ovarian cancer treatment, and its efficacy should be further validated through animal studies.

## 5. Conclusions

Our study provides compelling evidence that thymoquinone exerts significant apoptotic and antiproliferative effects on OVCAR-3 by modulating the RAS/RAF/MEK/ERK signaling pathway. This modulation leads to a reduction in key proliferation markers (Ki-67, Cyclin D1) and an upregulation of apoptotic markers (Caspase-3, Bax, Cleaved PARP), ultimately shifting the cellular balance toward apoptosis. Importantly, our findings indicate that the combination of thymoquinone with doxorubicin enhances cytotoxicity and apoptosis, suggesting a potential synergistic therapeutic effect against ovarian cancer.

Beyond its apoptotic role, thymoquinone demonstrated strong antioxidant properties, effectively reducing oxidative stress and preventing tumor progression. By neutralizing reactive oxygen species, TQ not only induces apoptosis but also inhibits metastatic potential, highlighting its dual action in cancer therapy. Additionally, wound healing and migration assays confirmed that the combination of TQ and DX significantly suppressed cell migration, reinforcing the potential of TQ in preventing cancer metastasis.

This study represents a novel insight into the mechanistic action of TQ against ovarian adenocarcinoma, particularly in combination with conventional chemotherapeutic agents. Given its ability to modulate key oncogenic pathways, inhibit proliferation, promote apoptosis, and reduce oxidative stress, thymoquinone emerges as a highly promising candidate for targeted ovarian cancer therapy. Future in vivo studies and clinical investigations will be essential to further establish TQ as a potential adjuvant or alternative therapeutic agent in combating ovarian cancer, offering new avenues for improved treatment strategies.

## Data Availability

All data supporting the findings of this study are available in public databases from PubChem, STRING and within the paper.
